# Microglia – mediated immunity partly contributes to the genetic association between Alzheimer’s disease and hippocampal volume

**DOI:** 10.1016/j.bbi.2019.02.011

**Published:** 2019-07

**Authors:** T.M. Lancaster, M.J. Hill, R. Sims, J. Williams

**Affiliations:** a UK Dementia Research Institute, School of Medicine, Cardiff University, UK; b Cardiff University Brain Research Imaging Centre (CUBRIC), School of Psychology, Cardiff University, UK; c MRC Centre of Neuropsychiatric Genetics & Genomics, School of Medicine, Cardiff University, UK

**Keywords:** Hippocampus, Alzhiemer’s disease, Microglia, Risk profile score, Polygenic, GWAS, MRI

## Abstract

Genome-wide association studies (GWAS) suggest that Alzheimer’s disease (AD) is partly explained by a burden of risk alleles (single nucleotide polymorphisms; SNPs) with relatively small effects. However, the mechanisms by which these loci cumulatively confer susceptibility remain largely unknown. Accumulating evidence suggests an association between increased AD risk allele burden (measured via a polygenic risk profile score; AD-RPS) with reduced hippocampal volume (HV) across a number of independent cohorts. These lines of research suggest that the reduced HV may be a causal mechanism of risk in the development of late-onset Alzheimer’s disease (AD). However, as RPS assesses broad, cumulative genetic risk, little is known about the biological processes which may explain this observation. Here, we leverage GWAS data from i) 17,008 late onset AD cases & 37,154 controls and ii) hippocampal volume (N = 12,147; N = 9707) to explore putative pathways that may explain this association. We first demonstrate an association between whole genome AD-RPS and HV (PT < 0.5, Z = −2.07, P = 0.038), confirming previous associations. Second, we restrict our analysis to SNPs within AD genes within a microglia mediated immunity network (N_GENES_ = 56). A microglia AD-RPS was further associated with HV (PT < 0.01; Z = −2.152, P = 0.031). Last, using a competitive, permutation based approach, we show that the common variation within this candidate gene-set is associated with HV, controlling for SNP set-size (P = 0.024). Together, the observations suggest that the relationship between AD and HV is partially explained by genes within an AD-linked microglia mediated immunity network.

## Highlights

• Alzheimer’s disease (AD) risk SNPs are linked to a reduction in hippocampal volume.
• A microglia mediated innate-immunity gene network may contribute to this association.
• Immunogenic-hippocampal plasticity SNPs (e.g NF-κB) could explain the relationship.

## 1 Introduction

Genome-wide association studies (GWAS) demonstrate that risk for Alzheimer’s disease (AD) is partly explained by a large number of single nucleotide variants with relatively small effects (Lambert et al., 2013, Sims et al., 2017). The cumulative burden of these risk allele can be estimated via risk profile score (RPS) analysis. Emerging evidence suggests that the combined effect of the AD-RPS may influence AD via risk mechanisms such as reduced cognitive ability (Del-Aguila et al., 2018, Ge et al., 2018, Louwersheimer et al., 2016, Mormino et al., 2016), increases in AD – related histopathology (Louwersheimer et al., 2016, Mormino et al., 2016) and other physical health markers (Hagenaars et al., 2018). However, the biological mechanisms by which genetic risk for AD confer susceptibility remains relatively unknown. Genetic neuroimaging studies have recently shown that the cumulative impact of AD risk alleles are associated with markers of brain health such as cortical (Li et al., 2018, Sabuncu et al., 2012) and hippocampal morphometry (Axelrud et al., 2018, Biffi et al., 2010, Foley et al., 2017, Lupton et al., 2016, Mormino et al., 2016). These observations suggest that the genetic architecture of AD may overlap with genetic variation that influences individual variability in hippocampal volume, a hypothesis that is supported by a recent GWAS of hippocampal volume (Hibar et al., 2017). Risk alleles for AD may confer risk of a smaller hippocampus throughout the lifespan (which provides reduced resilience in later life), supported by observations between AD – RPS and hippocampal volume in early adulthood (Foley et al., 2017, Mormino et al., 2016). These AD risk alleles may also associate with an accelerate trajectory of hippocampal atrophy (Harrison et al., 2016).

These studies use an AD-RPS that is estimated using a large number of alleles across the entire genome. Therefore, the biological pathways that underpin these putative associations remain elusive. A recent AD-GWAS suggests that common and rare genetic variation that confers risk may cluster within a network of molecules that coordinate microglia – mediated innate immunity (Sims et al., 2017). Preliminary evidence suggest that AD-RPS estimated via AD risk alleles within genes that play a role in immunity may contribute to peripheral markers of AD (Morgan et al., 2017). However, little work has explored the relationship between AD –linked genes with immune function and *in vivo* makers of brain health linked to AD, such as hippocampal volume (HV).

In the current study, we aim to test the hypothesis that AD-linked single nucleotide variants within the recently identified gene-network that orchestrates microglia – mediated innate immunity may partly explain the emerging relationship between AD and hippocampal volume. We perform this analysis using GWAS summary statistics from i) the latest AD GWAS (Lambert et al., 2013) and ii) recent GWAS of hippocampal volume (Elliott et al., 2018, Hibar et al., 2015). Our first objective is to replicate the association between whole genome AD-RPS and HV. Our second objective is to assess whether the microglia gene-network linked to AD contributes to this putative association.

## 2 Methods & materials

### 2.1 Samples

Polygenic score calculations were performed according to the procedure first described by the International Schizophrenia Consortium (International Schizophrenia et al., 2009). Training data were from the International Genomics of Alzheimer’s Project consortium that comprises 17,008 AD cases and 37,154 control subjects (Lambert et al., 2013). These data are publicly available from http://www.pasteur-lille.fr/en/recherche/u744/igap/igap_download.php. Hippocampal volume GWAS data were downloaded from the Enhancing Neuroimaging Genetics through Meta-Analysis (ENIGMA) GWAS analysis of subcortical volumes, available at http://enigma.ini.usc.edu/research/download-enigma-gwas-results/ and comprised of 13,163 individuals (Hibar et al., 2015). A replication data set was also acquired by averaging summary statistics derived from left and right hippocampal volume GWAS in UK Biobank (Image Derived Phenotype IDs: 2667 & 2682), available at http://big.stats.ox.ac.uk/download_page, comprising of 9707 individuals (Elliott et al., 2018). All GWAS data was corrected for demographic and genetic confounds.

### 2.2 Microglia-mediated innate immunity network selection

To explore the putative impact of AD risk alleles linked to microglia –mediated innate immunity, we restrict our AD-RPS and set-based analysis to loci within genes previously established via protein – protein interaction analysis (Sims et al., 2017). Briefly, this 56 gene network was created by protein-protein interaction analysis of gene modules enriched for variants associated with AD that were previously derived from brain co-expression networks (International Genomics of Alzheimer's Disease, 2015). We note that although this SNP set included genetic variants spanning all genes within the network, it did not include the rare variants identified via exome sequencing as AD effect sizes were only available for common risk alleles (minor allele frequency (MAF) > 0.01) and did not include the top hits in *PLCG2* and *ABI3*.

### 2.3 Genotype quality control and Alzheimer’s disease risk profile score (RPS) creation

For the AD-RPS, SNPs in the 1000 Genomes Project (phase 3) were used as reference data. Variants within both the major histocompatibility complex MHC (chr 6: 26,000–34,000 kb) and *APOE* (chr 19: 44,400–46,500 kb) regions were removed from the genotype data, as previously indicated (Tansey et al., 2018). In all cases, AD polygenic risk scores (RPS) were created using PRSice v1.25 risk profile software (Euesden et al., 2015) using a stringent clumping procedure [clump.p1 = 0.5, clump.p2 = 0.5, clump.kb = 300 kb, clump.r2 = 0.05] to remove correlated AD risk alleles. HV summary statistics were processed/quality controlled as previously outlined (Elliott et al., 2018, Hibar et al., 2015). A meta-analysis of beta coefficients (for both samples; ENIGMA & UKBB) was performed using the ‘metafor’ in R (Viechtbauer, 2010).

### 2.4 Competitive set analysis

In order to ascertain whether microglia SNP set size was contributing to putative explained variance, the relationship between AD linked microglia –mediated immune gene network and hippocampal volume was validated using two approaches. First, permutation analysis was conducted to set an empirical threshold by creating AD-RPS from 1000 permuted SNP sets (Cabrera et al., 2012) controlling for both SNP set size and IGAP significance. Second, gene set analyses in MAGMA v1.06 (de Leeuw et al., 2015) was used to test microglia gene-set enrichment. Briefly, common SNP association P-values were combined into gene-wide P-values (via the MAGMA SNP-wise mean model), using a window of 35 kb upstream and 10 kb downstream of each gene in order to include SNPs within regulatory regions (Network and Pathway Analysis Subgroup of Psychiatric Genomics, 2015). Only protein-coding genes were included in the analysis (N = 17,970). The gene set analysis method was used to perform a linear regression of gene-wide association against a gene-level for the meta-analysed (ENIGMA & UKBB HV) data. This analysis was two-tailed and corrected for gene size and SNP density.

## 3 Results

### 3.1 Whole genome AD-RPS, microglia AD-RPS and hippocampal volume

**Fig. 1:**
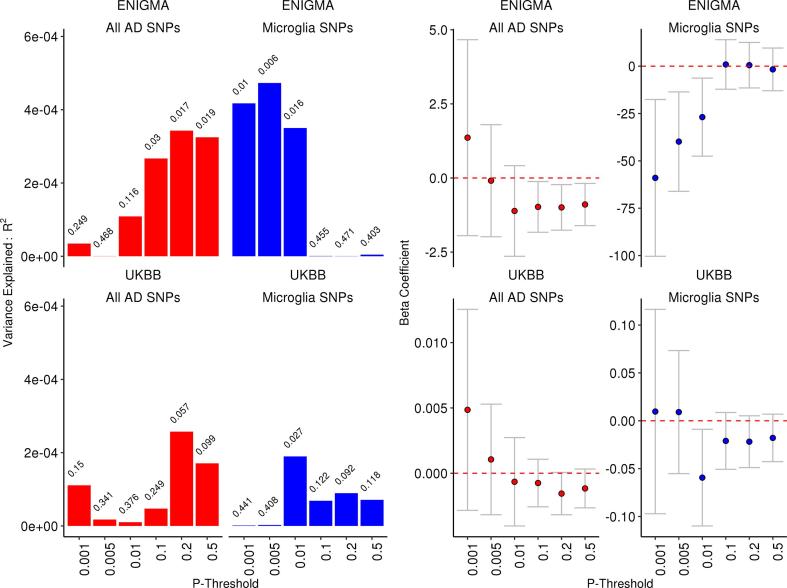
All AD-RPS (SNPs across whole genome) regressed on hippocampal volume (across whole genome; red) and microglia AD-RPS (SNPs within 56 microglia-mediated immunity genes (Sims et al., 2017; blue)). Left Y-axis = *R*^2^; Right Y axis = beta coefficients (+/− 95% confidence), X-axis = P-Threshold of AD-RPS. All AD-RPS are performed after the removal of the *APOE* and *MHC* loci. P values are annotated above each bar that denotes variance explained (R^2^) at each AD-RPS/P – threshold.

Consistent with prior reports, we first observed a relationship between total AD-RPS and hippocampal volume (Fig. 1; all AD-RPS, in red). This association was significant across multiple P thresholds (PT < 0.1–PT < 0.5; p-values above coloured bars). Including the *APOE* region slightly improved the model fit (PT > 0.05, P = 0.035; PT > 0.5, P = 0.007). We further observed a trend for association in our replication cohort (UKBB: PT < 0.2–0.5, P = 0.057–0.099). We then proceeded to restrict the AD-RPS to SNPs within the 56 genes within the identified microglia network and re-run the analysis. The microglia AD-RPS consisted of substantially less SNPs (Table 1), although explained a similar amount of variance in HV (across a range of P thresholds; PT < 0.001–PT < 0.01) (Fig. 1; variance explained coded in blue). We also observed this trend in our replication sample (PT < 0.01, P = 0.027). The putative enrichment of the microglia AD-RPS is empirically tested in 3.2: Competitive microglia set-based testing. SNPs that were associated the microglia AD-RPS in both samples (PT < 0.01) were present in 32/33 of the 56 AD-linked microglia-mediated immunity genes, many of which contained more than one independent variation (R^2^ < 0.05) that contributed to the AD-RPS (see 3.3 gene annotation). An analysis for AD-RPS (minus microglia SNPs) did not significantly affect any of these observations.

**Table 1:** Meta-analysis for association between AD-RPS and hippocampal volume (HV) across a progressive series of P-thresholds (PT > 0.001–0.5). All AD SNPs represents an AD-RPS derived from common SNPs (MAF > 0.01) across the whole genome (excluding *APOE* and *MHC* region). Microglia loci represents all common SNPs located in proximity to 56 genes implicated in the microglia – meditated innate immunity network. SNPs represents the number of AD associated risk variants considered at each P-threshold. Results in bold reflect significant effects in the meta-analysis.

	ENIGMA (N = 13163)		UKBB (N = 9707)		Meta-analysis (Z/P)			
PT	All AD SNPs	Microglia SNPs	All AD SNPs	Microglia SNPs	All AD SNPs		Microglia SNPs	
0.001	1479	16	1598	16	0.679	0.497	**−2.344**	**0.019**
0.005	5159	33	5723	34	−0.079	0.937	**−2.495**	**0.013**
0.01	8742	60	9755	61	−1.198	0.231	**−2.152**	**0.031**
0.1	45,698	221	52,308	234	−1.876	0.061	0.111	0.911
0.2	70,810	307	81,407	326	**−2.128**	**0.033**	0.070	0.944
0.5	117,446	473	136,598	529	**−2.070**	**0.038**	−0.249	0.804

### 3.2 Competetive microglia set-based testing

**Fig. 2:**
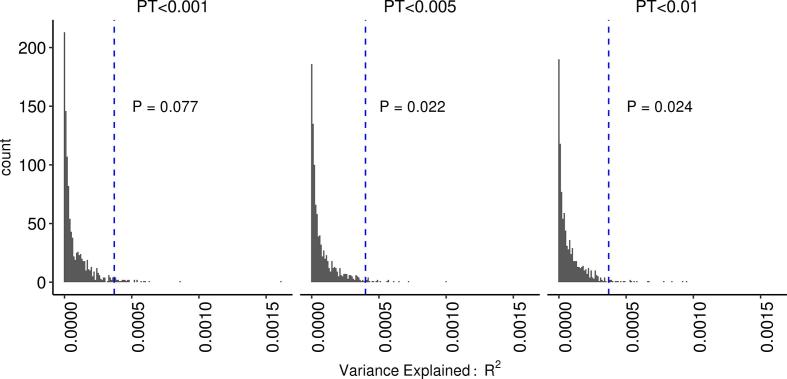
Variance explained (R^2^) by microglia AD-RPS (SNPs within 56 microglia-mediated immunity genes (denoted by blue dashed line) compared to 1000, comparably sized random AD RPS SNP sets at P-thresholds where microglia AD-RPS was significant. P value of enrichment at dashed line represents number of random AD-RPS that surpass microglia AD-RPS divided by total number of random AD-RPS permutations.

To ascertain whether the microglia AD-RPS was predictive of hippocampal volume above and beyond the set size, we drew a distribution of R^2^ from 1000 AD-RPS of randomly drawn IGAP SNPs. The microglia AD-RPS explained more variance (R^2^) in hippocampal volume compared to similar sized (number of SNPs) AD-RPS, randomly drawn SNPs from IGAP summary statistics (see Fig. 2). Gene set analysis was also conducted to ensure that the gene –set was associated with HV, controlling for number of SNPs in the model. For gene-wide competitive testing, 52 of the 56 genes were included in the final analysis, where 4 genes were excluded (*C3AR1, INPP5D, PTPN6, IKZF1*) for missing / incomplete SNP data. The AD-linked microglia gene set was significantly enriched for genes associated with hippocampal volume (N = 21,750, meta-analysed across ENIGMA & UKBB) compared to 17,970 background genes, correcting for gene size/density and sample size (β = 0.214 ± 0.0155, SE = 0.107, P = 0.023).

### 3.3 Gene annotation

**Fig. 3:**
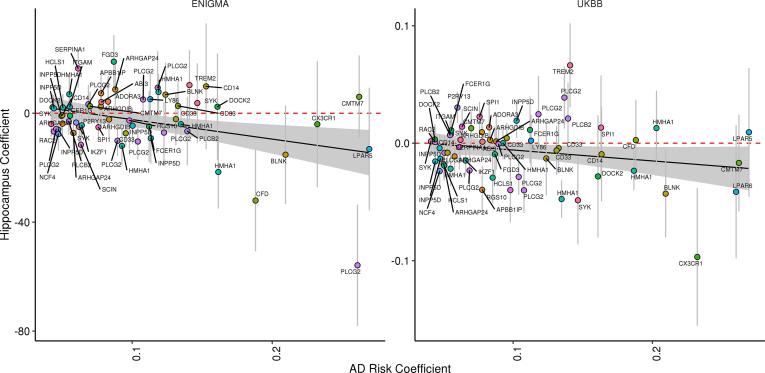
Diagnostic plots for microglia SNP sets for ENIGMA (N = 13,163; left: β = −26.9, P = 0.016, N_SNPS_ = 60) and UK Biobank (N = 9707; right: β = −0.05, P = 0.027, N_SNPS_ = 61), both constrained to the P-threshold < 0.01. Each SNP is plotted by coefficient in the risk score (x axis) versus estimated effect size for HV in the testing dataset (y axis). The solid black line shows the effect size estimate for the risk score on HV in each of the testing datasets. Grey bar represents 95% confidence intervals.

To assess the individual impact of SNPs used to estimate the microglia AD-RPS in both cohorts, we map each SNP to the nearest gene and plot each SNP effect size for both AD and HV (Fig. 3). The same 32 genes contributed to the association between microglia AD-RPS and HV in our discovery (ENIGMA) and replication (UKBB) sample. AD risk alleles with largest negative effect sizes for HV (intersected across samples) include SNPs within *PLCG2*, *BLNK, HMHA1, NCF4 & ARHGAP24* (top 5)*.*

## 4 Discussion

In the current study, we observed that whole genome AD-RPS was associated with hippocampal volume in a large discovery data set. This evidence suggests an overlapping genetic aetiology between Alzheimer’s disease (AD) and Hippocampal volume (HV) (Hibar et al., 2017). This observation supports previous studies demonstrating a relationship between AD-RPS and HV (Biffi et al., 2010, Chauhan et al., 2015, Foley et al., 2017, Lupton et al., 2016, Mormino et al., 2016). Critically, we further show that a smaller gene network (N_GENES_ = 56) supporting microglia-mediated innate immunity (as identified in a recent exome-wide study (Sims et al., 2017)) also show evidence for shared genetic overlap with HV, with a similar effect size to that of all AD risk alleles across the genome (excluding *APOE* and *MHC* regions). Post-hoc analysis suggested that multiple, independent loci within AD genes contributed to this association, several of which have previously been associated with HV such as *ABCA7* (Ramirez et al., 2016) and *CD33* (Wang et al., 2017). Furthermore, an intersection of effect sizes across samples demonstrated that the most influential SNPs were in genes such as *PLCG2*, *BLNK, HMHA1, NCF4 & ARHGAP24*. While it is difficult to elucidate individual SNP effects in a polygenic model, we note these genes may support biological plausible processes (such as *PLCG2-BLNK* interactions and NF-kappa B signalling – which has a key role in hippocampal plasticity (Albensi and Mattson, 2000, Meffert et al., 2003). Our observation is also supported by recent histological evidence showing that and AD-RPS (with and without *APOE*) is associated with microglia density exclusively within the temporal lobe (Felsky et al., 2018).

The comparable effect sizes for the whole genome AD-RPS and microglia AD-RPS demonstrate that simple, additive linear model of polygenic risk (i.e. whole genome risk profile scores) may not optimally capture the variance in a genetically linked phenotype, as biological specificity is not taken into account. Future studies that attempt to biological annotate risk profile scores should exercise caution as variance explained can vary as a function of discovery GWAS sample size, P-threshold criteria and weighted effect sizes (Dudbridge, 2013). In the current study, we attempted to control for the size of our candidate microglia AD-RPS SNP set size using competitive set-based testing. Our simulations suggest that the R2 of the microglia AD-RPS set was larger than chance, based on a distribution of 1000 randomly drawn AD-RPS.

We present these observations with the following considerations. First, we acknowledge that the impact of both genome-wide and microglia AD-RPS on hippocampal volume are small (<0.05% variance explained, in both cases). Second, as hippocampal volume was collected across a broad age range, we cannot infer whether these associations are fixed or dynamic across the lifespan. Hypothesis surrounding the impact of AD risk alleles on brain structure (and associated effect sizes) remain to be tested. Preliminary evidence suggests that while AD-RPS effects on hippocampal volume are present in young individuals (Axelrud et al., 2018, Foley et al., 2017), AD-RPS may influence the rate of age/AD related hippocampal atrophy (Harrison et al., 2016). We suggest that exploring putative dynamic effects of AD risk genes on brain structure across the lifespan are critical for understanding when these associations may occur. Taken together, we exercise caution of interpretation of effect sizes/variance explained by AD-RPS in the current study. Lastly, we solely explored a recently identified microglia-linked gene-set and suggest that future bioinformatics research should help to refine and uncover the principle biological gradients that underpin AD genetic risk (Tansey et al., 2018). This will help to delineate the various AD-linked process that may contribute to subcortical reductions in early and later-life processes. We also note that the AD-RPS component of our analysis was derived using reference data consisting of relatively common SNPs (MAF > 0.01) and did not include the rare SNPs identified via exome –based follow studies (Sims et al., 2017).

## 5 Conclusions

To conclude, the current study uses genome-wide summary datasets to confirm an association between AD risk alleles and hippocampal volume, with a smaller number of SNPs within a microglia mediated immunity network explaining a comparable amount of variance, above and beyond the size of the candidate SNP set. This provides the first evidence that AD genetic risk factors linked to microglia function may contribute to individual differences in brain health antecedent to the development of AD. Specifically, our analysis suggests immunogenic biological processes capable of influencing hippocampal plasticity, such as NF kappa B signalling. This study represents the one of the first pathway based approaches to explore shared genetic risk between AD and MRI phenotypes linked to the early makers of neurodegeneration and showcases the translational potential of pathway based polygenic approaches in imaging genetics. We suggest that dissecting biological pathways in AD and their biological correlates will further establish individual-orientated clinical strategies for early detection and intervention in AD.
